# The increased risk of sarcopenia in patients with cardiovascular risk factors in Suburb-Dwelling older Chinese using the AWGS definition

**DOI:** 10.1038/s41598-017-08488-8

**Published:** 2017-08-29

**Authors:** Peipei Han, Hairui Yu, Yixuan Ma, Li Kang, Liyuan Fu, Liye Jia, Xiaoyu Chen, Xing Yu, Lin Hou, Lu Wang, Wen Zhang, Haifang Yin, Kaijun Niu, Qi Guo

**Affiliations:** 1 Department of Rehabilitation Medicine, TEDA International Cardiovascular Hospital, Cardiovascular Clinical College of Tianjin Medical University, Tianjin, China; 2 Department of Rehabilitation Medicine, Tianjin Medical University, Tianjin, China; 3 Department of Cell Biology and Research Center of Basic Medical Science, Tianjin Medical University, Tianjin, China; 4 Nutritional Epidemiology Institute, Tianjin Medical University, Tianjin, China; 5 School of Public Health, Tianjin Medical University, Tianjin, China

## Abstract

The aim of the present study is to investigate the relationship between sarcopenia and cardiovascular risk factors (CVRF) in the Chinese elderly. A total of 1611 elderly individuals aged ≥60 years were enrolled in this study. The well-established CVRF of diabetes, hypertensions, and dyslipidemia were assessed. Sarcopenia was defined according to the recommended algorithm of the Asian Working Group for Sarcopenia (AWGS). Multiple logistic regression analyses and the linear regressions were used to evaluate the components of CVRF and the number of CVRF of elderly patients with sarcopenia. After adjusting for potential confounders, CVRF was associated with a high prevalence of sarcopenia in elderly Chinese populations. Furthermore, diabetes and hypertension, but not dyslipidemia, were found to be significantly associated with sarcopenia. The OR and 95% CI for sarcopenia of the participants with 1, 2, and 3 features of CVRF were 2.27(1.14–4.48), 4.13(1.80–9.46), and 4.90(1.01–23.81), respectively. A linear increase in the prevalence of sarcopenia was found to be associated with the number of CVRF components in the elderly population (*P* values for the trends < 0.001). Knowledge of known CVRF, particularly diabetes and hypertension, may help predict the risk for sarcopenia in the elderly.

## Introduction

Sarcopenia is a disease that is characterized by the decline of skeletal muscle mass, muscle strength, and physical performance^1^. It is associated with the aging process and can lead to significant morbidity and disability in elderly populations, including loss of independence, poor quality of life, and an increased risk of death^2–5^. The rate of sarcopenia in the elderly is expected to increase in the future^6^. This disease contributes to current increased health care costs^7^ and is becoming a major public health problem^8^. Because of the debilitating symptoms and increased healthcare cost burden, preventing and treating sarcopenia at an early stage is essential.

Many potential mechanisms of sarcopenia have been investigated such as decreased physical activity levels, hormone deficiencies, decreased dietary protein intake, and chronic inflammation accompanied by a production of catabolic cytokines^9^. Chronic inflammation and its resultant production of catabolic cytokines remains the most widely reported mechanism of sarcopenia. Chronic inflammation and catabolic cytokine production is a normal phenomenon linked to aging and is the major risk factor for age-related chronic diseases, such as diabetes^10^, hypertension^11^ and dyslipidemia^12^, all of which are well-known risk factors for cardiovascular disease (CVD)^13–15^.

CVD is the leading cause of death throughout the world and is increasing at an alarming rate in China^16^. The prevalence of its associated cardiovascular risk factors (CVRF) has increased exponentially in recent decades. To the best of our knowledge, only a few studies have been conducted to evaluate the association between sarcopenia and CVRF^17–22^, with no studies using the Asian Working Group for Sarcopenia (AWGS) definition of sarcopenia^23^. Of these studies, there were no clear consensus regarding the correlation between sarcopenia and CVRF. One study reported that sarcopenia was closely associated with increased risk factors for CVD^18^. Another study found sarcopenia to be weakly associated with predisposing risk factors for CVD in obese postmenopausal women^20^. Other papers have investigated the relationship between sarcopenia and components of CVRF, including diabetes, hypertension and dyslipidemia, but the results have not been clear cut. Moreover, few studies have surveyed the comparative significances of these features by the means of structural equation modeling.

Considering the absence of appropriate and consistent evidence regarding the association between sarcopenia and CVRF, it is necessary to study the relationship between sarcopenia and CVRF to improve public health in China, specifically in suburb-dwelling elderly populations. This study is particularly relevant because, as of 2012, more than 70% of Chinese elderly individuals live in suburban areas^24^.

## Results

Table 1 presents the clinical and anthropometric characteristics of participants by their sarcopenia status. Subjects with sarcopenia had a statistically significant higher age, but lower BMI, ASM, ASM/height^2^, gait speed, and grip strength compared with participants without sarcopenia (*P* < 0.05). Of the female and widowed individuals, the prevalence of illiteracy and smoking was much higher in the study group with sarcopenia compared with those without sarcopenia (*P* < 0.05). Diabetes was more prevalent in the sarcopenic participants relative to the nonsarcopenic participants (*P* < 0.05).

**Table 1:** Clinical, anthropometric characteristics of study subjects according to the presence of sarcopenia.

Parameter	Non Sarcopenia (n = 634)	Sarcopenia (n = 77)	*P*-value
Age (y)	66.33 ± 5.52	72.71 ± 7.58	< 0.001
Male, n (%)	320(50.5%)	29(37.7%)	0.040
BMI (kg/cm^2^)	25.55 ± 3.46	22.35 ± 2.91	< 0.001
ASM (kg)	19.66 ± 4.30	14.17 ± 3.36	< 0.001
ASM/Height^2^ (kg/cm^2^)	7.21 ± 0.97	5.67 ± 0.93	< 0.001
Grip strength (kg)	28.39 ± 9.09	19.45 ± 6.19	< 0.001
4-meter walking test (m/s)	1.02 ± 0.19	0.83 ± 0.19	< 0.001
IPAQ (Met/wk)	2079(865–2079)	1680(1826–3272)	0.083
Widowed, n (%)	83(13.1%)	28(36.4%)	< 0.001
Living alone, n (%)	90(14.2%)	16(20.8%)	0.126
Illiteracy, n (%)	131(20.7%)	30(39.0%)	0.001
Farming, n (%)	534(84.2%)	69(89.6%)	0.214
Smoking, n (%)			0.020
Never	304(47.9%)	24(31.2%)	
Former	117(18.5%)	18(23.4%)	
Current	213(33.6%)	35(45.5%)	
Alcohol drinking, n (%)			0.421
Never or Former	383(60.4%)	52(67.5%)	
<7 d/wk	84(13.2%)	7(9.1%)	
Daily	167(26.3%)	18(23.4%)	
CVRF			
Diabetes, n (%)	62(9.8%)	18(23.3%)	< 0.001
Hypertension, n (%)	267(42.1%)	36(46.8%)	0.437
Dyslipidemia, n (%)	186(29.3%)	25(32.5%)	0.570

ASM, appendicular skeletal muscle mass; BMI, body mass index; IPAQ, international physical activity questionnaire; Met/wk, metabolic equivalent task minutes per week; CVRF, cardiovascular risk factors.

The associations between the different CVRF components and sarcopenia are presented in Table 2. After adjusting for covariates in Models 1–3, the CVRF components, including diabetes and hypertension (but not dyslipidemia), were found to be significantly associated with sarcopenia (*P* < 0.05). The OR and 95% CI in the adjusted model 3 for the factors that were statistically significantly associated with sarcopenia were 2.66(1.41–5.01) for CVRF, 4.55(2.19–9.47) for diabetes and 1.82(1.02–3.27) for hypertension.

**Table 2:** Multiple logistic regression analysis of presence and components of CVRF for elderly patients with sarcopenia.

Variables	Univariate				Model 1				Model 2				Model 3			
	Coefficients	R2	OR (95%CI)	*P*-value	Coefficients	R2	OR (95%CI)	*P*-value	Coefficients	R2	OR (95%CI)	*P*-value	Coefficients	R2	OR (95%CI)	*P*-value
Presence of CVRF	0.424	0.008	1.53(0.92–2.55)	0.105	0.903	0.357	2.47(1.33–4.57)	0.004	0.906	0.359	2.47(1.34–4.58)	0.004	0.978	0.368	2.66(1.41–5.01)	0.003
**Components of CVRF**																
Diabetes	1.035	0.029	2.82(1.56–5.07)	0.001	1.381	0.367	3.98(1.94–8.17)	<0.001	1.407	0.371	4.08(1.98–8.41)	<0.001	1.515	0.380	4.55(2.19–9.47)	<0.001
Hypertension	0.188	0.002	1.21(0.75–1.94)	0.473	0.544	0.344	1.72(0.97–3.05)	0.062	0.540	0.347	1.72(0.97–3.04)	0.064	0.598	0.354	1.82(1.02–3.27)	0.046
Dyslipidemia	0.147	0.001	1.16(0.70–1.92)	0.570	0.385	0.340	1.47(0.81–2.68)	0.209	0.385	0.343	1.47(0.80–2.69)	0.211	0.375	0.348	1.45(0.79–2.66)	0.225

Notes: Model 1: adjustment for age, gender, and BMI; Model 2: Model 1+adjustment for marital status, educational level; Model 3: Model 2+adjustment for smoking, IPAQ, and peptic ulcer.

**Figure 1:**
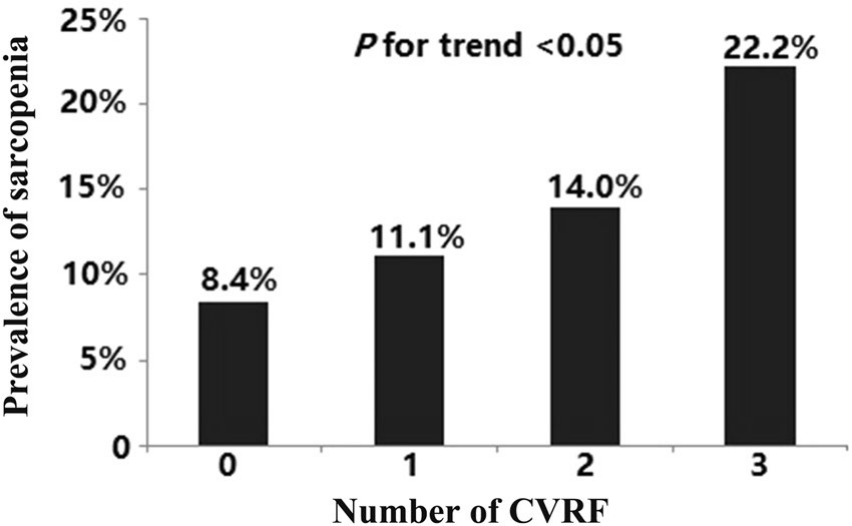
Prevalence of sarcopenia with respect to the number of CVRF.

The outcomes of the applications of the models that tested the effects of the increasing numbers of CVRF constituents on sarcopenia are illustrated in Table 3. There was a significant linear decrease in the risk of sarcopenia with an increasing numbers of CVRF constituents. After further covariate adjustment in Model 3, the OR and 95% CI of sarcopenia participants with 1, 2, and 3 features of CVRF were 2.27(1.14–4.48), 4.13(1.80–9.46), and 4.90(1.01–23.81) (*P* values for the trends < 0.001), respectively. The prevalence of sarcopenia increased steeply with numbers of CVRF (Fig. 1). Finally, we assessed the performance and calibration of all models. Model performance and calibration were consistently good in all models (Area under the ROC curves > 0.80; Hosmer-Lemeshow goodness of fit *P* > 0.05).

**Table 3:** Regression analysis of number of CVFR for elderly patients with sarcopenia.

Number of CVRF	Univariate			Model 1			Model 2			Model 3		
	Coefficients	OR (95%CI)	*P*-value	Coefficients	OR (95%CI)	*P*-value	Coefficients	OR (95%CI)	*P*-value	Coefficients	OR (95%CI)	*P*-value
1	0.307	1.36(0.78–2.38)	0.283	0.749	2.12(1.09–4.12)	0.028	0.749	2.11(1.09–4.12)	0.028	0.814	2.27(1.14–4.48)	0.020
2	0.579	1.78(0.92–3.47)	0.089	1.330	3.78(1.68–8.49)	0.004	1.345	3.84(1.71–8.62)	0.001	1.418	4.13(1.80–9.46)	0.001
3	1.137	3.12(0.95–10.25)	0.061	1.432	4.19(0.85–20.68)	0.079	1.430	4.18(1.05–20.63)	0.049	1.588	4.90(1.01–23.81)	0.041
*R2*	0.014			0.363			0.367			0.376		
*P* for trend	< 0.026			< 0.001			< 0.001			< 0.001		

Notes: Model 1: adjustment for age, gender, and BMI; Model 2: Model 1+adjustment for marital status, educational level; Model 3: Model 2+adjustment for smoking, IPAQ and peptic ulcer.

## Discussion

In the present study, we examined the effects of the prevalence of different numbers of CVRF components on sarcopenia using the AWGS definition in suburb-dwelling populations of elderly persons aged 60 and older in China. We found that the prevalence of sarcopenia was significantly proportional to the number of CVRF components. In particular, diabetes and hypertension were significantly associated with a higher risk of sarcopenia in the adjusted models. To our knowledge, our study is the first study that uses the AWGS definition to demonstrate a positive correlation between the prevalence of sarcopenia and CVRF constituents in China.

The major purpose of this study was to test the hypothesis that sarcopenia is associated with CVRF independent of other confounding factors in elderly Chinese populations. With similar findings to our study, a recent study by Kim *et al*.^18^ showed that sarcopenia was closely associated with increased risk factors of CVD. However, another study found that sarcopenia seems to be associated with lower risk factors of CVD in obese, postmenopausal women^20^. These conflicting results are likely due to the variable limitations related to the assessment of sarcopenia and CVRF, the differences between study populations, and varying ethnic characteristics. Asians have different CVD patterns compared to Caucasians. Koreans immigrants to the United States have significantly higher rates of stroke and lower rates of heart disease compared to Caucasians born in the United States^25^. Moreover, Asians have a higher risk for diabetes at a lower BMI compared to Europeans^26^, which means that diabetes and its related CVD risks might be different between these two populations. In particular, the results of the present study may be beneficial for further research on the relationship between sarcopenia and CVRF in elderly Chinese populations, which has not been previously studied.

In the present study, diabetes was associated with an elevated risk of sarcopenia, independently of age, gender, BMI, marital status, educational level, and smoking. This result was consistent with the results of previous studies^10, 27^. Kim *et al*.^10^ also reported that diabetes was associated with an increased risk of sarcopenia. Leenders *et al*.^28^ found that both appendicular skeletal mass and leg extension strength was lower in individuals with type 2 diabetes mellitus compared with normoglycemic controls. The mechanism of diabetes and its association with accelerated reduction of muscle mass and strength remains unclear. However, it has been reported that diabetes accelerates the loss of muscle mass and strength due to hyperglycemia, insulin resistance, endocrine changes, or the release of inflammatory cytokines^29^. Chronic hyperglycemia increases advanced glycation end products (AGEs). AGEs accumulate in skeletal muscle and cartilage and increase stiffness in patients with diabetes. It was reported that elevated AGEs were associated with poor grip strength in older women with moderate to severe disability and slow walking speed in older community-dwelling adults^30, 31^. Furthermore, inflammatory cytokines, such as tumor necrosis factor and interleukin 6, have detrimental effects on muscle mass, strength, and physical performance in older adults^32^. Based on the above, we postulate that diabetes impairs muscle mass, strength, and physical performance which leads to sarcopenia. These findings highlight the importance of diabetes management in the prevention of the onset of sarcopenia.

In this study, although univariate analysis showed no correlation between sarcopenia and hypertension, a multivariate statistical analysis revealed that hypertension was the 2nd most significant feature that was associated with sarcopenia after adjusting for the confounding factors age, gender, BMI, marital status, educational level, and smoking, which is consistent with previous findings^11^. To investigate whether sarcopenia is associated with hypertension, Han *et al*.^11^ surveyed 2,099 males and 2,747 females in older Koreans, and found that subjects with sarcopenia had a higher prevalence of hypertension than subjects without sarcopenia. The research on the relationship between of sarcopenia with hypertension is very few and limited, and the underlying mechanism that links sarcopenia and hypertension remains unclear. We hypothesize that during the aging process, mast cells, and inflammatory agents such as cytokines (e.g. TNF-alpha), advanced glycosylation products, and matrix metalloproteinases, appear to be related to a state of chronic low-grade inflammation. Recent studies have suggested that these inflammatory agents impair blood flow by damaging the microvascular endothelium^11^. As previously mentioned, inflammatory cytokines have detrimental effects on the bodies of older adults and may contribute to sarcopenia. Further studies are warranted to elucidate the causal relationship between hypertension and sarcopenia.

Only a few studies have reported the association between sarcopenia and dyslipidemia in Asia^12, 33^. Our study showed that there was no correlation between dyslipidemia and sarcopenia. However, in Baek *et al*.^33^ study, they investigated the association between sarcopenia and dyslipidemia in elderly Koreans and found that sarcopenia was associated with an increased risk for dyslipidemia. Besides, in a 4-year longitudinal study of 538 elderly Japanese, Kim *et al*.^12^ also found dyslipidemia was associated with sarcopenia. The literature regarding sarcopenia and dyslipidemia is few and complex. These contradictory results are likely due to variable susceptibilities related to studied population. In our study, we examined the relatively healthy participants with excluding patients of CVD; Baek *et al*. and Kim studied participants with general population. The studied populations of these researches vary greatly and cause a wide variation in the rate of sarcopenia^34^. Besides that, this confounding may also due to different proportions of patients receiving treatment is possible. Statin cholesterol lowering agents are known to have immunomodulatory effects, including inhibiting inflammation^35^. However, as already stated, inflammation is associated with increased risk of muscle mass and strength loss and cause sarcopenia. So, there is reason to suspect that statin cholesterol lowering agents may offset some of the dyslipidemia effects on sarcopenia. Thus, disparate results could occur if statin use varied between cohorts.

To our knowledge, the majority of studies have only focused on each individual component of CVRF: diabetes, hypertension and dyslipidemia. There is limited information on the association between the totality of CVRF components and the prevalence of sarcopenia. We note a positive correlation between the prevalence of sarcopenia and the increasing numbers of CVRF constituents. Although there is no previous research showing significant evidence between sarcopenia and the increasing total number of CVRFs directly, Brinkley *et al*.^36^ analyzed associations between physical function and inflammatory biomarkers in older adults across multiple comorbidities. Brinkley *et al*. demonstrated that elevated CRP and IL-6 levels are associated with poorer physical function in older adults with various comorbidities. Therefore, we believe the presence of comorbid diseases, such as diabetes, hypertension and dyslipidemia, elevate CRP and IL-6 levels and contribute to sarcopenia.

This study has a number of strengths. First, this study is one of the first studies using the AWGS criteria to identify the correlation between the prevalence of sarcopenia and CVRF constituents. Secondly, the study is also the first study to examine a uniquely defined group: suburban older men and women living in a discrete geographical area. Our participants were recruited from a suburban area and were leading a more physically active lifestyle, which may differ from subjects in other geographical areas.

Despite our efforts, some limitations exist. First, this study used a cross-sectional design, so it is not possible to determine causal relationships. Second, all participants in the present study were relatively healthy as we did not include participants who were unable to participate in the free annual national physical examination (i.e., those bedridden or with serious diseases). Due to these exclusions, our results might in fact underestimate the prevalence of sarcopenia and its associated health impact. A longer follow-up period with higher total number of CVD risk factors may provide a more definitive answer. Third, the self-reported variables may influence the precision of estimation. But similar to our study, some previous studies also collected the data by participants’ self-reporting. Thus, we suggest that it would not overturn the findings observed in our study. At last, the use of BIA for muscle mass assessment presents a drawback mainly due to hydration problems often observed in older persons—these may result in an underestimation of body fat and an overestimation of fat-free mass. BIA is not good standard, but it is well correlated with magnetic resonance imaging predictions and DXA. Besides, BIA is inexpensive, rapid, noninvasive, radiation-free, and convenient and is thus considered to be a portable alternative to DXA.

## Conclusion

In summary, we examined the associations between sarcopenia, each individual CVRF component, the total number of CVRF components in Tianjin, China, using the AWGS definition. We found that sarcopenia is independently associated with CVRF and in particular is associated with diabetes and hypertension. The prevalence of sarcopenia increased steeply with an increasing total number of CVRF. Therefore, the prevention and treatment of CVRF may be useful in preventing and delaying the onset of sarcopenia. A further prospective study is needed to establish the causality of CVRF components and sarcopenia.

## Methods

### Ethics statement

This research was approved by the Ethics Committee at Tianjin Medical University, and the methods were carried out in accordance with the principles of the Declaration of Helsinki. All records were anonymized and no individual information can be identified.

### Subjects

Our study population included residents from three areas: Hougu, East Chadian, and West Chadian of Tianjin, China. A total of 1166 older individuals in these areas joined the national free physical examination program from March 2013 to August 2015; all subjects were invited to participate in a comprehensive geriatric assessment. Of the 1166 subjects aged ≥ 60 years, participants who had previous diagnoses of CVD (i.e., myocardial infarction, coronary insufficiency, stroke, peripheral artery disease, and heart failure) (n = 252) were excluded from the study. Individuals with incomplete anthropometric measurements (n = 52) or laboratory test results (n = 151) were also excluded. A total of 711 subjects (349 men and 362 women) were included in the final statistical analysis. All participants provided informed consent prior to participation.

### Questionnaire

Data regarding sociodemographic and behavioral characteristics were obtained in a similar manner to our previous studies via face-to-face interactions^27, 37, 38^. Sociodemographic variables, including age, gender, marital status, educational level, and occupation were assessed. The assessed social history included smoking habits (never, former smoker, or current smoker) and drinking habits (never, former drinker, occasional drinker, or everyday drinker). Former smoker and current smokers were asked how many cigarettes they smoke per day and how long they had smoked^39^. We next obtained and assessed physical activity levels. The physical activity level was assessed using the short form of the International Physical Activity Questionnaire (IPAQ)^40^. A history of physical illness was established using standardized criteria that combined information from a questionnaire regarding history of a physical illness, a physician’s diagnosis, and the corresponding medication or current or past medical treatment.

### Analysis of blood samples and blood pressure

A blood sample was obtained from the antecubital vein from patients who fasted overnight for at least 10 h. After collection, samples were centrifuged for 15 min at 3000 rpm. The fasting plasma glucose (FPG), total cholesterol (TC), triglycerides (TG), high-density lipoprotein cholesterol (HDL-C), and low-density lipoprotein cholesterol (LDL-C) were measured using the Roche Modular P (Roche Diagnostic Company, Swiss)^37^. The systolic blood pressure (SBP) and diastolic (DBP) blood pressure were measured from the upper left arm using a sphygmomanometer after 10 min of sitting^13^.

### CVD risk factors assessment

Based on previous research^41^, the three CVD major risk factors were defined as follows: diabetes as the use of hypoglycemic agents, a self-reported history of diabetes, or a FPG ≥ 7.0 mmol/L or more^37^; hypertension as resting SBP ≥ 140 mmHg and/or DBP ≥ 90 mmHg and/or the self-reported current treatment for hypertension with antihypertensive medication^42^; dyslipidemia as using lipid-lowering drugs or having one or more of the following: TG ≥ 1.7 mmol/L, TC ≥ 5.18 mmol/L, HDL-C < 1.04 mmol/L, and LDL-C ≥ 3.37 mmol/L^43^.

### Assessment of Sarcopenia

Sarcopenia was defined according to the AWGS criteria^23^, in which a person who has low muscle mass, low muscle strength, and/or low physical performance was identified as having sarcopenia. Low muscle mass was classified as a relative skeletal muscle mass index (ASM/height^2^) less than 7.0 kg/m^2^ and 5.7 kg/m^2^ in men and women, respectively; low muscle strength was defined as a grip strength < 26 kg or < 18 kg for males and females, respectively; low physical performance was defined as a walking speed < 0.8 m/s for both males and females.

Muscle mass was measured using a direct segmental multi-frequency bioelectrical impedance analysis (BIA) (In-Body720; Biospace Co., Ltd,Seoul, Korea). Muscle strength was assessed by grip strength, measured using a dynamometer (GRIP-D; Takei Ltd, Niigata, Japan). Usual walking speed (m/s) on a 4-meter course was used as an objective measure of physical performance. Details of measurement methods have been described in our previous cross-sectional study^27^.

### Statistical analysis

Physical activity levels were reported as medians ± 25–75th percentiles for IPAQ; all other continuous variables were presented as means ± SD; classification variables were reported as percentages. Differences in baseline characteristics according to sarcopenia status were analyzed using t-tests, Pearson’s chi-square test, and Kruskal–Wallis rank tests. The associations between sarcopenia and CVRF were determined by analyzing each individual CVRF component or the total number of CVRF components. The simple logistic regression analysis was used to examine the independent influence of CVD risk factors on sarcopenia; the odds ratio (OR) and 95% (Confidence Interval, CI) were computed. In addition, adjustments for potential confounders such as age, gender, and BMI (model 1), marital status and educational level (model 2), and smoking, IPAQ, and peptic ulcer (model 3) were performed using multiple logistic regression analyses (Wald backward stepwise method). Analyses were repeated to determine the effect of individual CVRF components on sarcopenia components. The analysis generated regression coefficients, confidence intervals, Nagelkerke R2. Besides, the area under the receiver operating characteristic (ROC) curve and the Hosmer-Lemeshow goodness of fit statistic were calculated to assess the performance and calibration of the model, respectively. The *P*-values for the trend tests were determined by treating the number of CVRF components as a continuous variable (1–3) in order to observe the associations between an increase in the number of CVRF components and sarcopenia. All the statistical analyses were performed using SPSS version 19.0, and P-values less than 0.05 were considered statistically significant.

## References

[1] Delmonico MJ (2009). Longitudinal study of muscle strength, quality, and adipose tissue infiltration. Am J Clin Nutr..

[2] Woodrow G (2009). Body composition analysis techniques in the aged adult: indications and limitations. Curr Opin Clin Nutr Metab Care..

[3] Narici MV, Maffulli N (2010). Sarcopenia: characteristics, mechanisms and functional significance. Br Med Bull..

[4] Goodpaster BH (2006). The loss of skeletal muscle strength, mass, and quality in older adults: the health, aging and body composition study. J Gerontol A Biol Sci Med Sci..

[5] Cawthon PM (2007). Frailty in older men: prevalence, progression, and relationship with mortality. J Am Geriatr Soc..

[6] Landi F (2016). Serum levels of C-terminal agrin fragment (CAF) are associated with sarcopenia in older multimorbid community-dwellers: Results from the ilSIRENTE study. Exp Gerontol..

[7] Marcell TJ (2003). Sarcopenia: causes, consequences, and preventions. J Gerontol A Biol Sci Med Sci..

[8] Curcio F (2016). Biomarkers in sarcopenia: A multifactorial approach. Exp Gerontol..

[9] Schrager MA (2007). Sarcopenic obesity and inflammation in the InCHIANTI study. J Appl Physiol (1985)..

[10] Kim TN (2010). Prevalence and determinant factors of sarcopenia in patients with type 2 diabetes: the Korean Sarcopenic Obesity Study (KSOS). Diabetes Care..

[11] Han K (2014). Sarcopenia as a determinant of blood pressure in older Koreans: findings from the Korea National Health and Nutrition Examination Surveys (KNHANES) 2008-2010. PLoS One..

[12] Kim H (2015). Incidence and predictors of sarcopenia onset in community-dwelling elderly Japanese women: 4-year follow-up study. J Am Med Dir Assoc..

[13] Yu D (2009). Association between prehypertension and clustering of cardiovascular disease risk factors among Chinese adults. J Cardiovasc Pharmacol..

[14] Murakami, Y., Okamura, T., Nakamura, K., Miura, K. & Ueshima, H. The clustering of cardiovascular disease risk factors and their impacts on annual medical expenditure in Japan: community-based cost analysis using Gamma regression models. *BMJ Open*. **3** (2013). 10.1136/bmjopen-2012-002234 PMC3612762 23503577

[15] Cooper RS, Ordunez P, Iraola Ferrer MD, Munoz JL, Espinosa-Brito A (2006). Cardiovascular disease and associated risk factors in Cuba: prospects for prevention and control. Am J Public Health..

[16] Yang ZJ (2012). Prevalence of cardiovascular disease risk factor in the Chinese population: the 2007–2008 China National Diabetes and Metabolic Disorders Study. Eur Heart J..

[17] Abe T (2012). Influence of severe sarcopenia on cardiovascular risk factors in nonobese men. Metab Syndr Relat Disord..

[18] Kim JH, Cho JJ, Park YS (2015). Relationship between sarcopenic obesity and cardiovascular disease risk as estimated by the Framingham risk score. J Korean Med Sci..

[19] Sanada K (2010). A cross-sectional study of sarcopenia in Japanese men and women: reference values and association with cardiovascular risk factors. Eur J Appl Physiol..

[20] Aubertin-Leheudre M, Lord C, Goulet ED, Khalil A, Dionne IJ (2006). Effect of sarcopenia on cardiovascular disease risk factors in obese postmenopausal women. Obesity (Silver Spring)..

[21] Stephen WC, Janssen I (2009). Sarcopenic-obesity and cardiovascular disease risk in the elderly. J Nutr Health Aging..

[22] Park SH (2013). Sarcopenic obesity as an independent risk factor of hypertension. J Am Soc Hypertens..

[23] Chen LK (2014). Sarcopenia in Asia: consensus report of the Asian Working Group for Sarcopenia. J Am Med Dir Assoc..

[24] National Bureau of Statistics of China. Statistical communiqué on the 2012 national economy and social development of People’s Republic of China. *China Statistics*. (2013).

[25] Stellman SD (1996). Proportional mortality ratios among Korean immigrants to New York City, 1986–1990. Yonsei Med J..

[26] Chan JC (2009). Diabetes in Asia: epidemiology, risk factors, and pathophysiology. JAMA..

[27] Han P (2015). Prevalence and Factors Associated With Sarcopenia in Suburb-dwelling Older Chinese Using the Asian Working Group for Sarcopenia Definition. J Gerontol A Biol Sci Med Sci..

[28] Leenders M (2013). Patients with type 2 diabetes show a greater decline in muscle mass, muscle strength, and functional capacity with aging. J Am Med Dir Assoc..

[29] Morley JE, Malmstrom TK, Rodriguez-Manas L, Sinclair AJ (2014). Frailty, sarcopenia and diabetes. J Am Med Dir Assoc..

[30] Dalal M (2009). Elevated serum advanced glycation end products and poor grip strength in older community-dwelling women. J Gerontol A Biol Sci Med Sci..

[31] Semba RD, Bandinelli S, Sun K, Guralnik JM, Ferrucci L (2010). Relationship of an advanced glycation end product, plasma carboxymethyl-lysine, with slow walking speed in older adults: the InCHIANTI study. Eur J Appl Physiol..

[32] Visser M (2002). Relationship of interleukin-6 and tumor necrosis factor-alpha with muscle mass and muscle strength in elderly men and women: the Health ABC Study. J Gerontol A Biol Sci Med Sci..

[33] Baek SJ (2014). Sarcopenia and sarcopenic obesity and their association with dyslipidemia in Korean elderly men: the 2008-2010 Korea National Health and Nutrition Examination Survey. J Endocrinol Invest..

[34] Shafiee G (2017). Prevalence of sarcopenia in the world: a systematic review and meta- analysis of general population studies. J Diabetes Metab Disord..

[35] Koba S (2011). Physical activity in the Japan population: association with blood lipid levels and effects in reducing cardiovascular and all-cause mortality. J Atheroscler Thromb..

[36] Kavalipati N, Shah J, Ramakrishan A, Vasnawala H (2015). Pleiotropic effects of statins. Indian J Endocrinol Metab..

[37] Brinkley TE (2009). Chronic inflammation is associated with low physical function in older adults across multiple comorbidities. J Gerontol A Biol Sci Med Sci..

[38] Zhang W (2014). Poor lower extremity function was associated with pre-diabetes and diabetes in older chinese people. PLoS One..

[39] Han P (2016). Incidence, Risk Factors, and the Protective Effect of High Body Mass Index against Sarcopenia in Suburb-Dwelling Elderly Chinese Populations. J Nutr Health Aging..

[40] Reck, M. *et al*. Smoking History Predicts Sensitivity to PARP Inhibitor Veliparib in Patients with Advanced Non-Small Cell Lung Cancer. *J Thorac Oncol* (2017). 10.1016/j.jtho.2017.05.006 28748826

[41] Jiang CQ (2009). Effect of physical activity strength on the diabetes mellitus prevalence in the elderly under the influence of International Physical Activity Questionnaire]. Zhonghua Liu Xing Bing Xue Za Zhi..

[42] Vivanco-Hidalgo RM (2017). People with epilepsy receive more statins than the general population but have no higher cardiovascular risk: results from a cross-sectional study. Eur J Neurol..

[43] 1999 World Health Organization–International Society of Hypertension Guidelines for the Management of Hypertension. Guidelines Sub-Committee. *Blood Press Suppl*. **1**, 9–43 (1999). 10401540

